# The Role of Matrix Metalloproteinase 13 and Vitamin D in Osteoarthritis: A Hospital-Based Observational Study

**DOI:** 10.7759/cureus.45437

**Published:** 2023-09-18

**Authors:** Purushottam Kumar, Santosh Kumar, Abhilasha Abhilasha, Akrity Singh, Uday Kumar

**Affiliations:** 1 Department of Biochemistry, Nalanda Medical College and Hospital, Patna, IND; 2 Department of Trauma and Emergency Medicine, Indira Gandhi Institute of Medical Sciences, Patna, IND; 3 Department of Biochemistry, Indira Gandhi Institute of Medical Sciences, Patna, IND

**Keywords:** vitamin d, elisa, knee osteoarthritis, kellgren-lawrence stage, matrix metalloproteinase 13

## Abstract

Introduction

Osteoarthritis (OA) is the most common form of degenerative joint disease characterized by the progressive degeneration of articular cartilage, osteophyte formation, and joint space narrowing. Matrix metalloproteinases (MMPs) are potential biomarkers for osteoarthritis.

Aims and objective

The study's aim is the estimation of serum and synovial fluid matrix metalloproteinase (MMP) 13 and serum vitamin D levels in the grade 3 and grade 4 stages of osteoarthritis according to the Kellgren and Lawrence (KL) system of classification.

Materials and methods

A total of 100 subjects were included; of them, 25 patients with grade 3 and 25 patients with grade 4 knee osteoarthritis diagnosed clinically and radiologically according to the Kellgren and Lawrence criteria have been enrolled in the study, and 50 patients with knee pain having a diagnosis other than degenerative OA of the knee were taken as controls. Venous blood and synovial fluid have been collected from all of them for the estimation of MMP-13 and vitamin D. The enzyme-linked immunosorbent assay (ELISA) and chemiluminescent microparticle immunoassay (CMIA) methods were used for the estimation of MMP-13 and vitamin D, respectively.

Results

The mean value of synovial fluid MMP-13 was found to be elevated in grade 4 as compared to grade 3 and the control group, whereas the mean value of serum MMP-13 was found to be elevated in grade 3 as compared to grade 4 and control. The level of serum vitamin D was found deficient in OA patients as compared to control. The Kruskal-Wallis test was performed to compare these groups, and there was a significant difference between these groups (p-value of <0.05).

Summary and conclusion

High synovial and serum MMP-13 is associated with knee structural abnormalities in patients with knee OA as compared to the control group suggesting that MMP-13 can be a biomarker in knee OA, whereas the decreased level of vitamin D may be associated with an increased risk for the progression of OA; hence, serum vitamin D may be a good indicator for the prediction of the initiation of OA.

## Introduction

Osteoarthritis (OA) stands as a prominent degenerative joint disease, predominantly affecting the elderly, characterized by joint pain, swelling, stiffness, and reduced mobility [1]. OA exacts a toll on patients' productivity, quality of life, and socioeconomic landscape, placing burdens on individuals, societies, and healthcare systems [2]. The foremost risk factor for OA is age; other factors such as traumatic knee injury, obesity, genetic predisposition, abnormal mechanical stress, and inflammation triggered by infection or surgery also contribute significantly [3].

OA's structural hallmarks include chronic inflammation, progressive articular cartilage degradation, and subchondral bone sclerosis, with irreversible articular cartilage degradation driving the disease's advancement [4]. Articular cartilage, devoid of blood vessels, is composed of extracellular matrix (ECM) and chondrocytes, distributed across four layers: superﬁcial, middle, deep, and calciﬁed cartilage zones [5]. Type II collagen stands as the matrix's primary structural protein [6], with its turnover rate, mediated by chondrocytes synthesizing matrix components and proteolytic enzymes breaking them down, being inherently sluggish [7]. This balance between synthesis and degradation is chiefly regulated by matrix metalloproteinases (MMPs) and endogenous tissue inhibitors of metalloproteinases [8].

MMP-13, a key enzyme, is pivotal in cleaving type II collagen, central to cartilage breakdown in OA-affected joints. As cartilage breakdown products outpace elimination, inflammation ensues, drawing T-cells to the synovial cavity and prompting type A synoviocyte activation. These stimulated synoviocytes churn out inflammatory cytokines and MMPs, such as tumor necrosis factor-alpha (TNF-α), interleukin (IL) 1, IL-6, and MMP-13, compounding the catabolic effects on chondrocyte metabolism, thereby hastening OA progression [9].

Risk factors such as mechanical stress and injuries may escalate the expression of both anabolic and catabolic factors. However, catabolic factors take precedence, leading to heightened MMP-13 secretion by chondrocytes, intensifying cartilage degradation. Cartilage breakdown products are released into synovial fluid, where synoviocytes engage in phagocytosis due to their phagocytic function [10].

Matrix metalloproteinases (MMPs), zinc-containing endopeptidases dependent on serum calcium for action [11], degrade various extracellular matrix proteins [12]. While MMP-13 is expressed during embryonic development to aid bone mineralization, it is overexpressed in pathological conditions such as carcinomas, rheumatoid arthritis (RA), and OA. Its preferred substrate, type II collagen, establishes MMP-13 as a key contributor to OA progression, aggressively breaking down this collagen type [13].

Vitamin D deficiency, a global concern, manifests through heightened osteoblastic activity and bone turnover due to low serum 25-hydroxyvitamin D (25-OH-D) levels [14]. Both OA and vitamin D deficiency are age-linked; OA worsens with age as vitamin D stores decline, especially in elderly females [15]. Ultimately, this leads to alterations in bone mineral metabolism, exacerbating osteoporosis and propelling OA progression [16].

The pathological progression of OA is still not well understood in spite of extensive research; therefore, we intend to study MMP-13 as a potential diagnostic marker for OA progression in this study.

## Materials and methods

The research design of our study was a hospital-based prospective observational study. A total of 100 cases were included in the study. In the case group, 50 diagnosed cases of degenerative osteoarthritis fulfilling both inclusion and exclusion criteria were included. In the control group, 50 patients with knee pain in the age group of 40-65 years, having a diagnosis other than degenerative OA of the knee, were taken as control. Patients of the osteoarthritis case group were categorized into two groups, moderate osteoarthritis group (Kellgren and Lawrence {KL} grade 3) and severe osteoarthritis group (KL grade 4), according to the Kellgren and Lawrence (KL) system for the classification of osteoarthritis. This study was conducted on patients attending the outpatient department (OPD) of the Department of Orthopedics of Indira Gandhi Institute of Medical Sciences, with complaints of knee pain. The investigations for MMP-13 in synovial fluid and serum vitamin D were performed in the Department of Biochemistry of Indira Gandhi Institute of Medical Sciences, Patna, after the approval of the Institutional Ethics Committee. The duration of the study was 23 months. A written informed consent was obtained from all the participants before participation in the study.

Inclusion criteria

Patients aged 40-65 years and of either sex coming with knee pain in the OPD with their written consent were included in the study. Patients having degenerative OA of the knee were diagnosed clinically and radiologically.

Exclusion criteria

**Figure 1:**
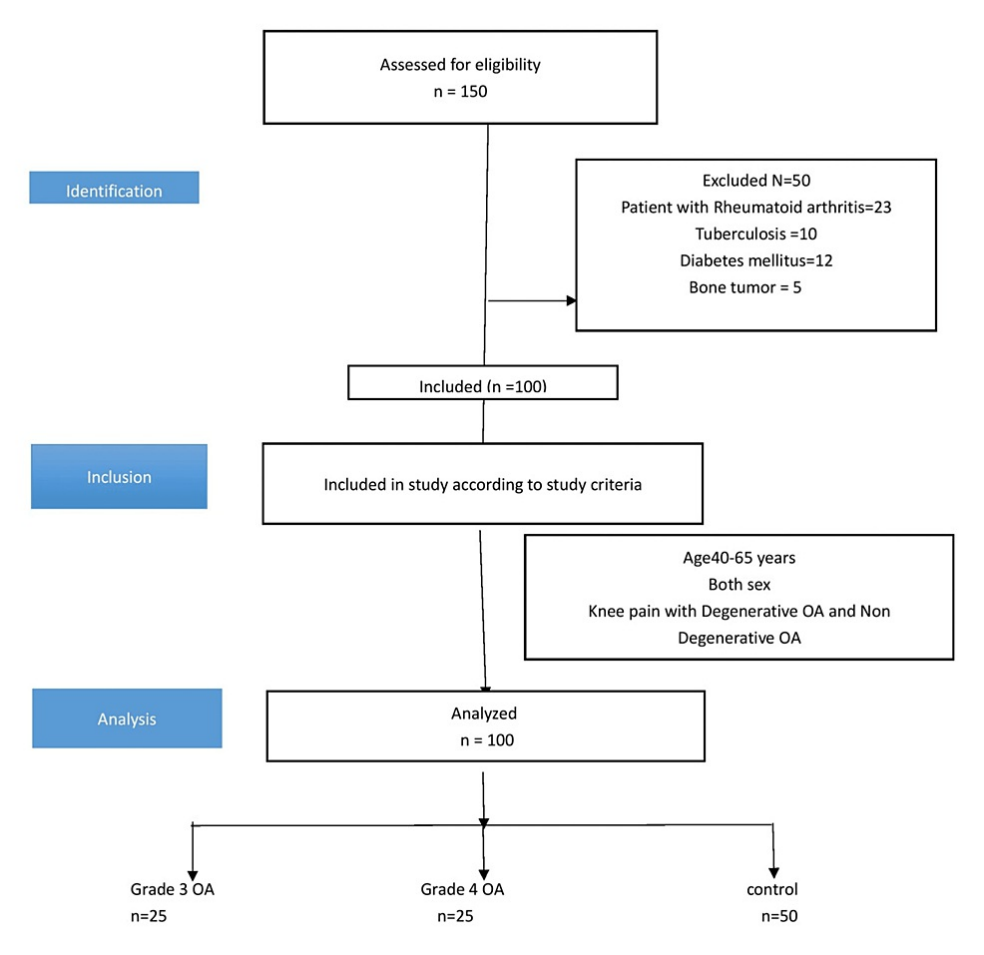
STROBE diagram STROBE, Strengthening the Reporting of Observational Studies in Epidemiology; OA, osteoarthritis

Patients with complaints of other inflammatory joint diseases such as rheumatoid arthritis (RA), tuberculosis, diabetes mellitus, and bone tumor were excluded from the study (Figure 1).

Synovial fluid collection

Patients were fully explained about the procedure and taken in confidence. Arthrocentesis was done under proper aseptic and antiseptic precaution. Synovial fluid was collected and centrifuged for 15 minutes at 1000 g and analyzed immediately or stored at 2°C-8°C for next-day analysis. The quantitative estimation of MMP-13 was done by enzyme-linked immunosorbent assay (ELISA).

Blood collection

After an overnight fasting, blood samples were collected from all the participants in the morning under full aseptic precautions. Patients were fully explained about the procedure, and 5 mL of collected blood was transferred in a plane vial. The blood vial was kept at room temperature for at least 30 minutes and allowed to clot before centrifuging it at 3000 rpm for 10 minutes. The centrifuged serum was then transferred by pipette into another tube. The clear serum was analyzed for the estimation of MMP-13 by ELISA and vitamin D by chemiluminescent microparticle immunoassay (CMIA) method.

Statistical analysis

All statistical analyses were done using the Statistical Package for Social Sciences (SPSS) 16.0 (SPSS Inc., Chicago, IL) for the Windows program. All data are expressed as means ± SD and median (interquartile range {IQR}), frequency, and percentages as required. Student's t-tests and chi-square (χ^2^) tests were used to compare means and proportions, respectively. The Kruskal-Wallis test was done to compare groups with more than two categories. To generate box plots and bar graphs, SPSS 16.0 and Windows Microsoft (MS) Excel 10.0 (Microsoft® Corp., Redmond, WA) were used. A p-value of less than 0.05 (two-tailed) was considered statistically significant.

## Results

The study group is composed of 100 patients; 50 patients with osteoarthritis fulfilling the inclusion and exclusion criteria and 50 controls were included in the study with their written consent. The demographic patterns of patients are shown in Table 1.

**Table 1:** Demographic profile of patients P-value of <0.001: highly significant (chi-square test) OA: osteoarthritis

Patient's profile		Case, n (%)		Control, n (%)	P-value
Sex	Male	15 (30)		31 (62)	
	Female	35 (70)		19 (38)	
Age group	≤50 years	14 (28)		33 (66)	<0.001
	>50 years	36 (72)		17 (34)	
Grade of OA	Grade 3 OA	≤50 years	9 (36)	-	0.208
		>50 years	16 (64)	-	
	Grade 4 OA	≤50 years	5 (20)	-	
		>50 years	20 (80)	-	

After detailed history and clinical evaluation, synovial fluid was taken for the estimation of MMP-13 and serum for the estimation of MMP-13 and vitamin D. There was significant difference between grade 3 and grade 4 osteoarthritis and control for all the parameters (Table 2) such as synovial MMP-13, serum MMP-13, and serum vitamin D.

**Table 2:** Comparison between grade 3 and grade 4 osteoarthritis and control for various parameters and their p-value *Kruskal-Wallis test, df = 2 MMP-13, matrix metalloproteinase 13; χ^2^, chi-square; df, degrees of freedom

Parameter	Grade 3 (n = 25) (mean ± SD)	Grade 4 (n = 25) (mean ± SD)	Control (n = 50) (mean ± SD)	χ^2^	P-value*
Synovial MMP-13 (pg/mL)	186.2 ± 78.2	238.2 ± 93.5	79.3 ± 29.4	45.508	<0.001
Serum MMP-13 (pg/mL)	117.6 ± 40.8	89.6 ± 36.2	49.1 ± 24.6	45.237	<0.001
Serum vitamin D (ng/mL)	11.4 ± 3.2	11.9 ± 4.0	20.4 ± 6.0	13.662	<0.001

**Figure 2:**
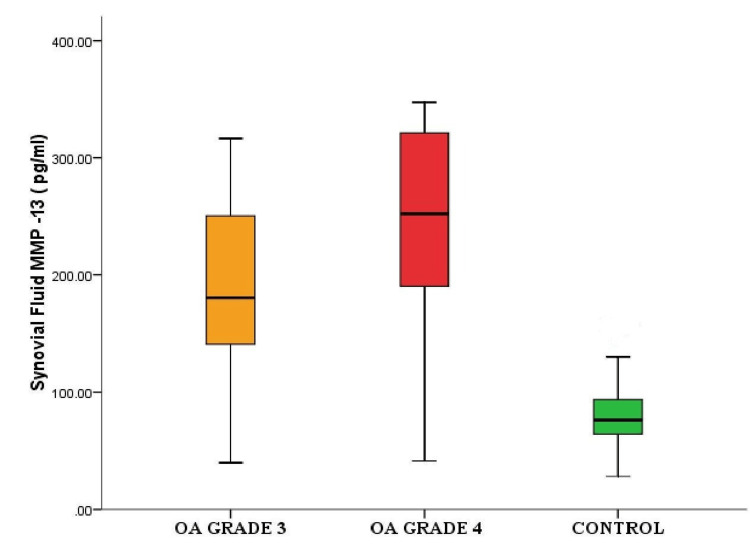
Box plot showing significant difference in the levels of synovial fluid MMP-13 in grade 3 osteoarthritis (OA), grade 4 OA, and the control group MMP-13: matrix metalloproteinase 13

There was a significant difference in the levels of synovial fluid MMP-13 in grade 4 OA compared to grade 3 OA as shown in Figure 2.

**Figure 3:**
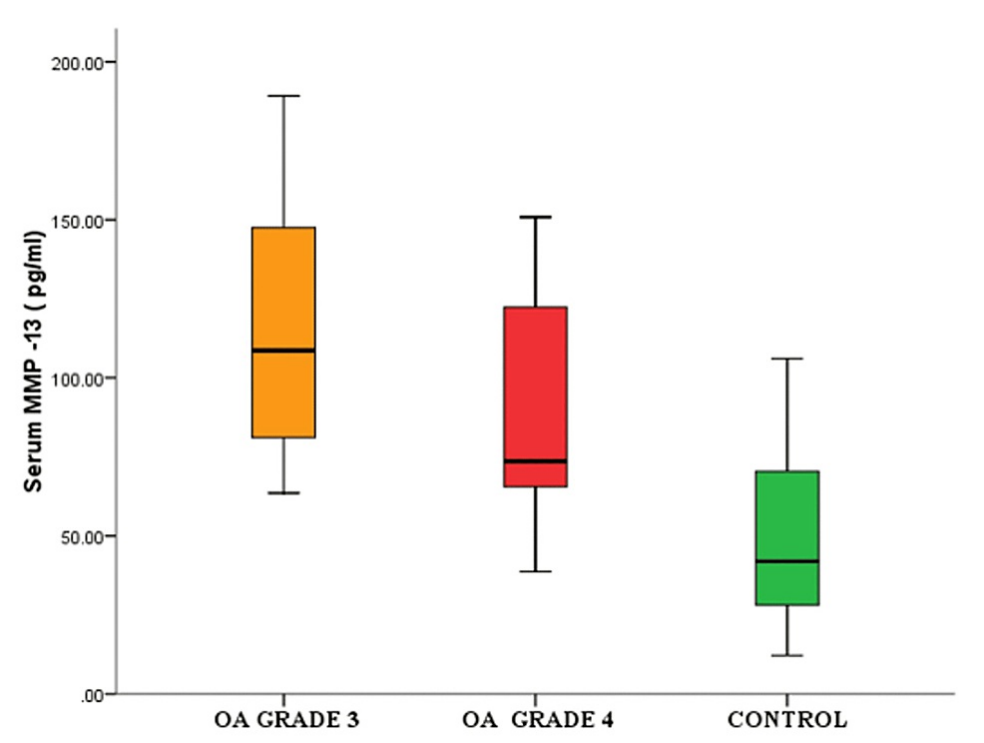
Box plot showing significant difference in the levels of serum MMP-13 in grade 3 OA, grade 4 OA, and the control group OA, osteoarthritis; MMP-13, matrix metalloproteinase 13

**Figure 4:**
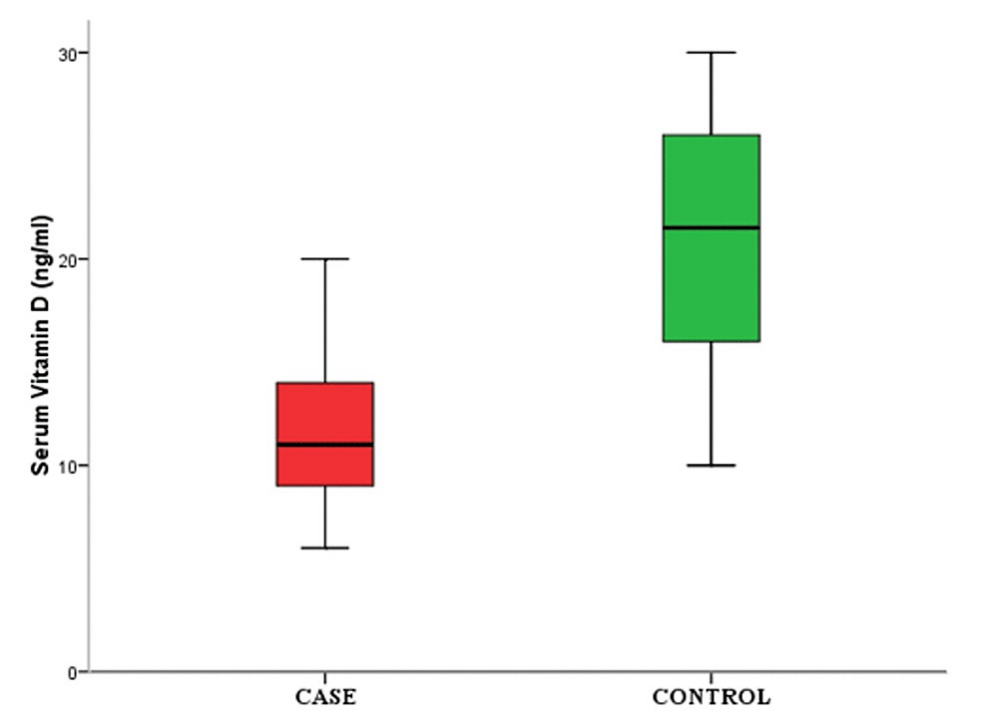
Box plot showing significant decrease in serum vitamin D level in cases compared to control group

There were a significant difference in the levels of serum MMP-13 in grade 3 OA compared to grade 4 OA as shown in Figure 3 and a decrease in the level of serum vitamin D in cases compared to controls (Figure 4).

## Discussion

The structural changes observed in OA are due to a combination of mechanical factors and biochemical pathways. In our study, there was a significant difference between synovial MMP-13 levels in the case and control groups. A similar result was observed for serum MMP-13 level, which was higher in cases compared to controls (Table 1 and Figures 1, 2). For grade 3 OA, the synovial fluid MMP-13 levels (186.2 ± 78.23 pg/mL) in comparison to serum MMP-13 levels (117.63 ± 40.82 pg/mL) were significantly higher (p-value = 0.001). In grade 4, the synovial fluid MMP-13 levels (238.16 ± 93.47 pg/mL) were also significantly higher than serum MMP-13 levels (89.61 ± 36.16 pg/mL) (p-value of <0.001). It was observed that the synovial fluid MMP-13 level increased as the OA progressed from grade 3 to grade 4, whereas the level of serum MMP-13 decreased in grade 4 as compared to grade 3.

Similar findings were suggested by Özler et al. (2016) [17], in which they found that in both grades 3 and 4, mean synovial fluid MMP-13 levels (4.31 ± 1.24 pg/mL) were found to be significantly higher compared to serum MMP-13 levels (1.089 ± 1.519 pg/mL) (p-value = 0.001). Compared to grade 3 (3.95 ± 4.45), grade 4 synovial fluid MMP-13 (4.76 ± 5.82) was elevated, whereas grade 3 OA serum MMP-13 (1.128 ± 0.308) was elevated compared to grade 4 OA (1.038 ± 0.204) (p-value = 0.438 and 0.430, respectively) [18].

Vitamin D has been shown to negatively correlate with the activity of metalloproteinase enzymes. In our study, low level of vitamin D and increased MMP-13 level in the osteoarthritic group were found (Table 1).

In our study, it was found that all the patients with osteoarthritis were vitamin D-deficient and the serum vitamin D levels were significantly lower in this group (mean value of 11.6 ± 3.6 ng/mL, as compared to the control group {mean value of control: 20.4 ± 6.0 ng/mL}) (p-value of <0.001) (Table 1 and Figure 3).

Glowacki et al. (2003) also found in their study that the prevalence of low vitamin D levels in patients with OA of the knee was 22% [19].

Lane et al. studied the baseline and follow-up of 237 subjects who obtained low levels of plasma 25-OH-D, which increased the risk of developing OA by approximately threefold. Of the patients with OA enrolled for his study, 88.57% were vitamin D-deficient, whereas 61.43% of controls have vitamin D in optimal range [20].

In our study, there was no significant difference in serum vitamin D levels between the different grades of OA.

Limitations

The sample size was modest, and it is possible that with a larger sample size, more significant associations can be detected. As it was a cross-sectional study, the causal relationships were unknown. This needs to be determined by future cohort studies. The subjects were recruited from the hospital rather than from the community randomly, so the results may not be generalizable to knee OA patients from the community.

## Conclusions

Osteoarthritis (OA) is one of the most common degenerative joint diseases with a complex mechanism. MMP-13-mediated regulation may improve or inhibit the onset of OA through altering the autophagy process and epigenetic modification. MMP-13-mediated OA involves numerous regulatory pathways, most of which are not known, and many of these pathways remain unknown. The onset and progress of OA through MMP-13-mediated regulatory mechanisms can be beneficial in the diagnosis and treatment. In the future, MMP-13 inhibitors may bring a breakthrough in the treatment of OA. High synovial and serum MMP-13 is associated with knee structural abnormalities in patients with knee OA as compared to the control group, suggesting that MMP-13 can be a biomarker in knee OA.

This study shows the high prevalence of vitamin D deficiency in patients with osteoarthritis. A decreased level of vitamin D may be associated with an increased risk for the progression of OA; hence, serum vitamin D may be a good indicator for the prediction of OA disease initiation. Hence, the quantification of serum vitamin D in OA may be used as an early biomarker. The findings of the present study provide an improved understanding of the early diagnosis and treatment of OA following the identification of various potential biomarkers, which can be used as a target for drug molecules in the future.
